# Quasi-extinction risk and population targets for the Eastern, migratory population of monarch butterflies (*Danaus plexippus*)

**DOI:** 10.1038/srep23265

**Published:** 2016-03-21

**Authors:** Brice X. Semmens, Darius J. Semmens, Wayne E. Thogmartin, Ruscena Wiederholt, Laura López-Hoffman, Jay E. Diffendorfer, John M. Pleasants, Karen S. Oberhauser, Orley R. Taylor

**Affiliations:** 1 Scripps Institution of Oceanography, University of California, San Diego, 9500 Gilman Drive, La Jolla CA 92093, USA; 2 United States Geological Survey, Geosciences and Environmental Change Science Center, Denver, CO 80225, USA; 3 United States Geological Survey, Upper Midwest Environmental Sciences Center, 2630 Fanta Reed Road, La Crosse, WI 54603, USA; 4 School of Natural Resources and the Environment and Udall Center for Studies in Public Policy, The University of Arizona, Tucson, AZ 85721, USA; 5 Department of Ecology, Evolution, and Organismal Biology, Iowa State University, Ames, IA 50011, USA; 6 Department of Fisheries, Wildlife and Conservation Biology, University of Minnesota, St Paul, MN, USA; 7 Department of Ecology and Evolutionary Biology, University of Kansas, Lawrence, KS, USA

## Abstract

The Eastern, migratory population of monarch butterflies (*Danaus plexippus)*, an iconic North American insect, has declined by ~80% over the last decade. The monarch’s multi-generational migration between overwintering grounds in central Mexico and the summer breeding grounds in the northern U.S. and southern Canada is celebrated in all three countries and creates shared management responsibilities across North America. Here we present a novel Bayesian multivariate auto-regressive state-space model to assess quasi-extinction risk and aid in the establishment of a target population size for monarch conservation planning. We find that, given a range of plausible quasi-extinction thresholds, the population has a substantial probability of quasi-extinction, from 11–57% over 20 years, although uncertainty in these estimates is large. Exceptionally high population stochasticity, declining numbers, and a small current population size act in concert to drive this risk. An approximately 5-fold increase of the monarch population size (relative to the winter of 2014–15) is necessary to halve the current risk of quasi-extinction across all thresholds considered. Conserving the monarch migration thus requires active management to reverse population declines, and the establishment of an ambitious target population size goal to buffer against future environmentally driven variability.

Monarchs are a charismatic species with high levels of public interest in their status and conservation^1^,^2^. In 2014, due to concern over the lowest overwintering population size since recordkeeping began in 1994, the U.S. Fish and Wildlife Service was petitioned to list the Eastern, migratory subpopulation (hereafter “population”) of monarchs as a threatened species under the Endangered Species Act^3^ and has subsequently initiated a status review to determine whether listing is warranted. More recently, the White House announced a strategic goal of increasing the Eastern population of the monarch butterfly to 225 million butterflies occupying an area of approximately 6 hectares in the overwintering grounds in Mexico by 2020 4.

Given the cultural significance of monarch butterflies and difficulty of addressing the causes of their decline, it is important to establish the extent of their vulnerability and identify the population size needed to reduce the risk of quasi-extinction to an acceptable level. Population viability analysis is a key input to decisions about whether or not to list a species as threatened or endangered and an important step in the process of conservation planning. Here we present a novel Bayesian multivariate auto-regressive state-space model to assess quasi-extinction risk. We do not attempt to specify an acceptable level of risk, but present a range of results bracketing the level likely to be adopted by decision makers. Throughout this paper we refer to “quasi-extinction” as the loss of a viable migratory population of monarchs in eastern North America.

The size of the monarch overwintering population has followed a general downward trend, with the lowest populations recorded in the last three censuses^5^ (Fig. 1). The cause of the recent decline has been predominantly attributed to the loss of breeding habitat, primarily in the U.S.^6^,^7^. Monarchs lay eggs on many species of milkweed (*Asclepias* spp.) that developing larvae require for food. Declines in milkweed abundance are well documented and highly correlated with the adoption of herbicide-tolerant genetically modified corn and soybeans^6^, which now constitute 89% and 94% of these crops, respectively, in the U.S.^8^. Other threats, summarized by Shahani *et al.*^9^, include habitat loss in the wintering sites, climate change, insecticides (including neonicotinoids and others), mowing regimes, invasive species, and disease incidence.

We created a multivariate first-order auto-regressive state-space model^10^,^11^ to generate population parameter estimates for use in quasi-extinction risk forcasting. We fit the model using both area (ha) of forest occupied by overwintering colonies (1993–2014)^5^ and total annual egg production in the Midwest (1999–2014)^6^. Our modeling approach permitted us to separate measurement error and process noise (population stochasticity due to biological and environmental variability) in these data and subsequently generate probabilistic quasi-extinction risk estimates for the population.

In the context of population viability analysis, estimates of process noise can dramatically influence extinction risk, as environmental variability can cause populations to stochastically hit quasi-extinction thresholds well before a deterministic decline would indicate; this is particularly true for small populations^12^. Measurement (observation) error at the overwintering sites is substantial, originating primarily from the difficulty in measuring the density of monarchs at each colony. Published density estimates vary widely, ranging from 6.9–60.9 million monarchs per hectare in the overwintering areas^13^,^14^. Similarly, we expect measurement error associated with egg production in the Midwest to be considerable, and independent from measurement error associated with the overwintering population. Our modeling approach thus separately estimates process noise and both measurement errors, and affords the ability to generate quasi-extinction probabilities based on probabilistic estimates of (1) process error (independent of measurement errors), (2) estimated overwintering population size in the last census year (winter 2014/2015), and (3) the growth rate of the population. Importantly, because these estimates are probabilistic, we were able to translate uncertainty in these parameter estimates into probabilistic estimates of quasi-extinction risk over specific time horizons.

## Results

**Figure 1:**
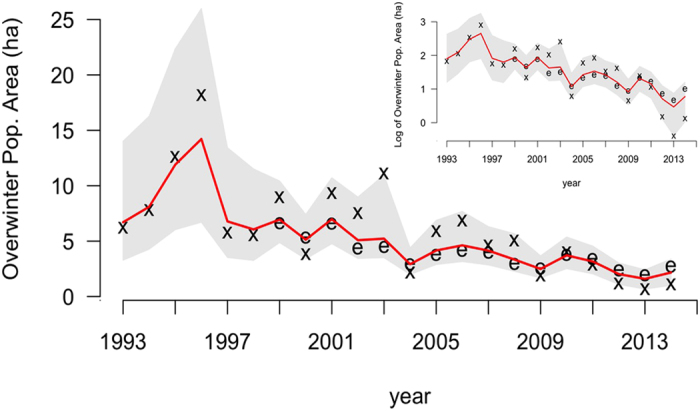
Model estimated annual over wintering population size (median of posterior estimates; red line) with 95% credible intervals (gray shaded area). The **x** symbols define overwintering habitat area data from Mexico, while the **e** symbols represent observations of annual egg production in the Midwest scaled to match the magnitude of the overwintering data (Extended Data Table 1). The inset depicts the data and model results on a log-scale.

**Figure 2:**
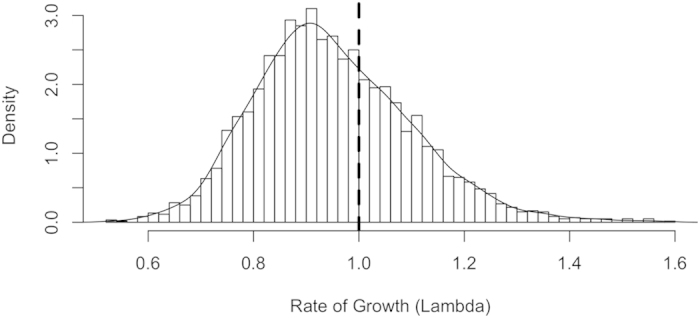
Posterior likelihood distribution for the estimated annual rate of growth in the monarch population. The vertical dashed line identifies the rate of growth that would result in no growth or decline from year to year (Lambda = 1). Lambda values >1 result in population growth, while values <1 result in decline.

Model results indicate that the monarch population declined by 84% from a population maximum of 13.90 ha (6.92 – 25.61; hereafter, median and 95% credible interval; CI) in the winter of 1996–97, to 2.20 ha (1.00–3.14 CI) in 2014–15 (the most recent survey year; Fig. 1). Over the modeled timeframe (1993–2014), the estimated annual rate of growth (λ) was 0.94 (0.69–1.3 CI), with 66% of the posterior distribution falling below λ = 1 (Fig. 2). In other words, based on the data and uninformative priors, there is a 66% chance the average annual growth rate underlying the stochastic trajectory of the population is below 1.

Our model estimated process noise (standard deviation) at 0.49 (0.28–0.80 CI), overwintering habitat area measurement error at 0.44 (0.21–0.67 CI), and egg production measurement error at 0.04 (0.001–0.41 CI). The variability in true population size ultimately drives quasi-extinction probability over short periods of time^15^. Our estimate of process noise is considerably higher than the range of values reported in the literature^16^, although to date no synthetic study has attempted to generate a range of plausible process noise values for insects in general or lepidopterans in particular. The apparent high process noise identified by our model reflects the fact that the population is subject to stochastic environmental events such as extreme temperatures or winter storms^14^,^17^; indeed, the susceptibility of insect populations to environmental stochasticity is widely accepted as the main driver of high variability in population size^17^. Additionally, the fact that monarchs undergo multiple generations between successive survey periods likely contributes to the high process noise in the time-series.

We used the model to estimate the probability of quasi-extinction over 10- and 20-year periods based on a range of quasi-extinction thresholds from 0.01–0.25 ha (see methods). Starting with the winter 2014/2015 estimated population level and using the average growth rate over the period of record, our model predicts the probability of quasi-extinction to be 3–42% over 10 years and 16–62% over 20 years (Table 1; Extended Data Figs 1 and 2). We also generated estimates of quasi-extinction risk over 10 or 20 years based on different starting population sizes, and under an assumed population growth rate of 1.0 (Table 2). We performed this latter latter exercise in order to assess quasi-extinction risk associated with different population size management targets. Scenario specific risk assessments assume that population size targets are met in the initial year, and that management actions have successfully mitigated declining trends in the population.

## Discussion

While monarchs are currently under consideration for listing as threatened under the Endangered Species Act (ESA), there is no existing convention for defining threatened or endangered status under the ESA based on a quantitative extinction risk analysis. Given that the annual cycle of the monarch population spans Mexico, the United States, and Canada, it is worth placing our quasi-extinction risk results in the context of international standards for species conservation status. The International Union for the Conservation of Nature (IUCN) Redlist criteria provides a set of conservation classifications ranging from Least Concern to Critically Endangered^18^. Using the median values of our quasi-extinction risk analysis, monarchs would be classified as Endangered according to the IUCN for all but the lowest extinction threshold, given that quasi-extinction risk over 20 years is greater than 20% regardless of the quasi-extinction threshold considered (Table 1).

Our finding of a high probability of quasi-extinction over the next two decades stands in stark contrast to the only other published monarch extinction risk estimate. Flockhart *et al.*^7^ used a spatially structured, stochastic and density-dependent periodic projection matrix model to estimate the cumulative probability of quasi-extinction (<1000 individuals) over the next century, which they reported to be ~5%. The difference between our quasi-extinction risk estimates is principally attributable to the quasi-extinction thresholds used, the incorporation of uncertainty in parameter estimation, and the way in which process noise was incorporated into forecasts. The quasi-extinction threshold of 1000 individuals used by Flockhart *et al.*^7^ is likely too optimistic. Based on an assumed density of 40 million monarchs/ha in overwintering habitat^13^,^14^, 1000 monarchs would occupy just 0.25 square meters of forest. We believe it is unrealistic to expect population functions to remain intact at such a reduced abundance for a species that clusters in winter for thermal regulation, needs to find mates during northward migration across the entire eastern part of North America, and is susceptible to extreme weather^14^,^19^. Simple population models with relatively few estimated demographic parameters, such as ours, generally yield more accurate estimates of future population states^15^,^20^. While our model does not account for density dependence, our estimates of quasi-extinction risk are likely robust, given that the population is in decline and has fluctuated widely^21^.

Our modeling exercise uses overwintering habitat area as a proxy of the population size of monarchs. However, the specific conversion between habitat area and the number of monarchs is uncertain. Previous studies^13^,^14^,^22^ attempting to estimate the density of monarchs per ha in overwintering grounds have generated disparate estimates, although arguably the best estimates of density with uncertainty come from Calvert^13^ who used multiple census techniques to arrive at an estimated uncertainty interval of 6.9–60.9 million monarchs ha^−1^. If we assume this interval represents a normally distributed 95% confidence interval, the error associated with the estimate is approximately 0.23 SD (after converting the interval to log ha by assuming a fixed monarch density of 40 million monarchs/ha). This is approximately half the estimate of measurement error associated with the log of overwintering habitat area occupied that we derived from our modeling exercise (0.44 SD). This difference is not surprising, however, given that the measurement error term in our model essentially represents both 1) the ability to accurately estimate the true number of ha occupied by monarchs, and 2) uncertainty in the number of monarchs per ha, which undoubtedly varies by year. In contrast, the estimate from Calvert^13^ represents only the latter uncertainty (# of monarchs ha^−1^) for a single year.

The selection of a target population size is a key step in conservation planning and requires wildlife managers to determine a level of risk that they are willing to accept. We calculated the risk of quasi-extinction over 10 and 20 years for starting population sizes ranging from 1–10 ha (Table 2). If attained, the near-term (2020) population target of 6 ha adopted by the White House will reduce the risk of quasi-extinction over 10–20 years by more than 50% for all thresholds considered. For all but the highest quasi-extinction threshold of 0.25 ha, reaching this goal would be sufficient to transition the population from Endangered to a lower-threat category under the IUCN criteria.

Our target population exercise indicates a high level of quasi-extinction risk over relatively short time windows, even when assuming large starting population sizes, which highlights the peril that monarchs currently face. Given the population’s present low numbers, poor reproductive success by monarchs in future breeding seasons due to weather conditions and reduced breeding habitat, followed by catastrophic mortality while overwintering in Mexico, could bring the monarch migration to the brink of extinction. Stabilizing the growth rate of the population and meeting the 2020 target population goal will substantially decrease extinction risk due to stochastic environmental processes. The documented decline in available breeding habitat^6^,^7^ is likely a major driver of the monarch population decline and suggests that efforts to recover the population towards the 2020 goal should focus on the creation and restoration of habitat.

## Methods

### Data

We used two different time series depicting dynamics of the Eastern, migratory population of monarch butterflies. First we used the log of the total extent (ha) of overwintering forest area occupied in Mexico per year from 1993–2014 measured by the World Wildlife Federation- Mexico and the Monarch Butterfly Biosphere Reserve (MBBR)^5^. Second, we used the log of the estimated total amount of egg production in the Midwest per year from 1999–2012^6^ and extended this through 2014 for the current analysis (Extended Data Table 1). The egg production per year was based on the average estimated eggs per milkweed stem for that year multiplied by the number of available milkweed stems on the landscape in that year. Eggs per stem estimates come from weekly monitoring data reported to the Monarch Larva Monitoring Project (MLMP - http://www.mlmp.org) from citizen scientists throughout the monarch range. We used the eggs per stem value from the week of peak egg production as an index for the production estimation. The number of available milkweed stems was based on estimates of the density of milkweeds in different habitats based on surveys^23^,^24^ and the area on the landscape occupied by those habitats using U.S. Department of Agriculture (USDA) databases^25^ and a function describing the decline of milkweeds in agricultural fields^6^.

### Estimating Population Parameters

We developed a multivariate first-order auto-regressive state-space model^10^,^11^,^12^ to generate monarch population parameter estimates for quasi-extinction risk forecasting. We chose a Bayesian modeling approach because the resulting parameter posterior distributions provide a complete characterization of parameter uncertainty that can be seamlessly propagated through to our quasi-extinction risk analysis. In so doing, we can account for uncertainty in quasi-extinction risk due to uncertainty in the parameters controlling population change. Moreover, the hierarchical nature of state-space modeling is easily handled through Bayesian estimation.

The log-scale population modeling framework takes the following form:


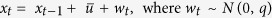



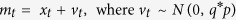



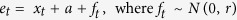


In the above set of equations, *x* represents the state process (estimated log of the true size of the overwintering population in Mexico) across all years *t* for which we have data. The state process evolves from one year to the next according to a mean population growth rate, 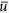, and associated random yearly deviates to growth, *w*_*t*_, which we assumed to be normally distributed with a mean of 0 and a standard deviation *q* (process noise). The exception to this is that we must directly estimate the population state in the first year (1993) using an uninformative uniform prior, since there is no prior year to evolve from. Note that 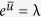, the average annual (non-logged) population growth rate, where λ values of <1 result in population decline, while values of >1 results in population growth.

Values of *m*_*t*_ are the log of yearly estimates of Mexican overwintering habitat occupied, which we assumed to deviate from the state *x*_*t*_ by *v*_*t*_. Values of *v*_*t*_ follow a normal distribution with a mean of 0 and a standard deviation of q^*^p (measurement error), where *q* is the process noise and *p* is a proportion parameter. We used this parameterization based on the assumption that process noise in the time series is greater than the measurement error associated with the Mexican overwintering data. This assumption is based on a consensus among the authors that measurement error is exceeded by variation in population sizes caused by demographic and environmental variation, and because process noise is typically the predominant form of variability in time series’ of wild populations^26^. As such, measurement error in *m* is defined to be a proportion (*p*) of process noise *q*.

Similarly, the log of annual estimates of Midwestern egg production, *e*_*t*_, are assumed to deviate from the state *x*_*t*_ by *a*, a scaling parameter^11^ that shifts the egg production index to the same scale as the overwintering habitat index, and *f*_*t*_, where values of *f* are assumed to be normally distributed with a mean of 0 and a standard deviation of *r* (measurement error). Note that, in the context of the linear modeling framework outlined above, the parameter *a* is essentially an intercept term.

We fit our model using R^27^ and JAGS^28^ (Just Another Gibbs Sampler), and assessed convergence by examining parameter trace-plots and calculating Gelman-Rubin diagnostics using the CODA^29^ package in R. Parameter estimates are provided in Extended Data Tables 1 and 2.

For the purposes of quasi-extinction risk forecasting, we report the following posterior estimates after removing variation caused by measurement error:

- *x*_2014_, the estimate of the true size of the Mexican overwintering population during the winter of 2014–15,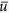, the monarch population growth rate, and
- *q*, the process noise associated with the monarch population.

The process noise, *q*, represents year-to-year variability in the population after accounting for overall trend through time (*ū*, the mean growth rate in log space) and after removing variation caused by measurement error. Note that using these model estimates, we can simulate the population forward in time from its estimated current size (*x*_2014_).

### Calculating Quasi-extinction Risk

Because each posterior draw from the Bayesian state-space model represents a complete set of likely parameter values, we can use all the posterior draw sets to generate annual probabilistic quasi-extinction risk estimates that account for both uncertainty in population parameters and uncertainty due to the stochastic population process (random yearly growth or decline due to process noise). For each posterior draw *i*, we simulate the population 20 years into the future 1000 times, starting at *x*_2014*i*_, using the growth rate 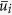 and process noise *q*_*i*_. For each of *i* simulation sets, we subsequently calculated the percent of runs that fall below a given quasi-extinction threshold (described below). Because we carried out this exercise for each *i* posterior draw, we can subsequently generate median and 95% credible interval estimates of quasi-extinction risk that account for population parameter uncertainty. Using a model run with a burn in of 4e5 iterations, and a sample window 10e5 iterations (3 separate chains) thinned by 600, our model achieved satisfactory convergence based on both visual inspection of trace plots and Gelman and Rubin diagnostics^30^; the Potential Scale Reduction Factors for all parameters were below 1.05.

The quasi-extinction threshold, or population size at which extinction of the Eastern monarch migration becomes inevitable, is unknown. We do know that monarch ecology exhibits at least two characteristics that suggest the likelihood of a strong Allee^26^ effect and therefore the existence of an extinction threshold: tightly clustered overwintering colonies convey important microclimate advantages that diminish as colony size decreases^19^,^31^ and the increased efficiency of locating mates in overwinter aggregations^32^,^33^,^34^. Diminishing colony size can therefore result in higher winter mortality rates and lower fecundity in the spring, which can cause the population growth rate to drop below replacement.

Regarding the size of winter aggregations specifically, expert opinion among the monarch biologist authors of this manuscript (Taylor, Oberhauser, Pleasants) favored an extinction threshold of no less than 0.05 ha, with most favoring a threshold closer to 0.25 ha. For reference, the smallest observed size of a Mexican overwintering colony is 0.01 ha^5^, and the minimum number of colonies that has been observed in Mexico in any given year is 7^5^. To be sure that we have captured the real quasi-extinction threshold, we opted to consider a range of values from 0.01 ha (1 viable colony, equivalent to approximately 1 occupied tree) to 0.25 ha. Our lowest (most optimistic) quasi-extinction threshold is therefore equivalent to the smallest observed colony size on record, which permits the loss of all but one core colony and the associated redundancy of multiple colonies before quasi-extinction occurs. The presence of multiple colonies provides a buffer against extinction during winter storms because of their variable storm severity within the overwintering region^14^. Our least optimistic estimate of the threshold suggests that quasi-extinction could occur well before the population declines to a single core colony at the minimum observed size. These values are intended to bookend the plausible range of the extinction threshold based on the best available information.

A mechanistic approach to estimating suitable threshold values was presented by Wells *et al.*^33^, who developed a model of monarch butterfly mating in California overwinter clusters that demonstrated a relationship between mating success and overwintering density. They found that reproductive success was highest for aggregations over 250,000 individuals. As aggregation size dropped below 250,000, reproductive success started to decline, and the rate of decline increased substantially below 50,000. They further noted that stable overwintering aggregations in California normally fall within this range (50,000–250,000), but acknowledge that they did not account for predation, which is a significant factor at Mexican overwintering sites and would likely require shifting this range higher. In addition, this model does not account for the population-level benefits of having multiple colonies.

The number of individuals present in overwintering colony areas is strongly dependent upon the density of monarchs per hectare. There are five published estimates of monarch overwintering densities, ranging from 6.9–60.9 million monarchs per hectare^13^,^14^. At the low end of this range, a quasi-extinction threshold of 0.05 ha would yield ~345,000 individuals, which may be just small enough to impact mating success according to the Wells *et al.*^33^ model, once higher Mexican predation rates are accounted for. A threshold of 0.01 ha yields just 69,000 individuals at the lowest density and ~324,000 at the average density, which is more firmly in the realm of reduced mating success according to the Wells *et al.*^33^ model.

### Estimating a target population size

We ran the quasi-extinction risk simulation across a range of initial starting population sizes and thresholds to develop associated quasi-extinction risks for each combination of values. The quasi-extinction risk simulation was run at initial population sizes ranging from 1 ha–10 ha, in 1 ha increments. We ran these simulations for quasi-extinction thresholds of 0.01, 0.05, 0.15, and 0.25 ha over 10 and 20 years. Because the intent of this exercise was to inform the selection of a recovery goal based on population sizes that confer protection against quasi-extinction risk, we conducted our simulation exercise under the assumption that population declines have been halted and the annual growth rate (λ) of the population is 1. All quasi-extinction events in our simulations are thus exclusively a function of process noise.

## Additional Information

**How to cite this article**: Semmens, B. X. *et al.* Quasi-extinction risk and population targets for the Eastern, migratory population of monarch butterflies (*Danaus plexippus*). *Sci. Rep.* **6**, 23265; doi: 10.1038/srep23265 (2016).

## Supplementary Material

Supplementary Information

## Figures and Tables

**Table 1:** Quasi-extinction risk (median probabilities with 95% credible intervals) over 10 & 20 years for a range of quasi-extinction thresholds, based on the current population growth rate (Lambda) of 0.94.

Time horizon	Quasi-extinction Threshold			
	0.01 ha	0.05 ha	0.15 ha	0.25 ha
10 years	**0.03** (0.00–0.34)	**0.13** (0.01–0.62)	**0.30** (0.04–0.81)	**0.42** (0.07–0.88)
20 years	**0.16** (0.01–0.83)	**0.34** (0.01–0.93)	**0.52** (0.06–0.98)	**0.62** (0.09–0.98)

**Table 2:** Quasi-extinction risk (median probabilities) over 10 and 20 years given different starting population sizes and quasi-extinction thresholds and based on a population growth rate of 1.0.

Quasi-extinction Threshold	Starting Population Size									
	1 ha	2 ha	3 ha	4 ha	5 ha	6 ha	7 ha	8 ha	9 ha	>10 ha
*10-Year Projection*										
0.01	0.02	0.01	0.01	0.00	0.00	0.00	0.00	0.00	0.00	0.00
0.05	0.13	0.06	0.04	0.03	0.02	0.02	0.01	0.01	0.01	0.01
0.15	0.30	0.18	0.13	0.10	0.08	0.06	0.06	0.04	0.04	0.04
0.25	0.42	0.26	0.19	0.15	0.13	0.11	0.09	0.08	0.07	0.06
*20-Year Projection*										
0.01	0.11	0.07	0.05	0.04	0.03	0.03	0.03	0.02	0.02	0.02
0.05	0.28	0.19	0.14	0.13	0.10	0.10	0.09	0.08	0.07	0.07
0.15	0.46	0.34	0.28	0.24	0.20	0.19	0.17	0.16	0.14	0.14
0.25	0.57	0.43	0.34	0.30	0.27	0.25	0.23	0.22	0.20	0.18

For reference, the most recent winter population size was measured at 1.13 ha.
