# Supplementary material for: Quasi-extinction risk and population targets for the Eastern, migratory population of monarch butterflies (Danaus plexippus)
Source: Sci Rep. 2016 Mar 21;6:23265. doi: 10.1038/srep23265 (PMC4800428; doi:10.1038/srep23265)
Supplement: Supplementary Information [file srep23265-s1.pdf]

Supplemental Information for:

Quasi-extinction risk and population targets for the Eastern, migratory population of monarch butterflies (*Danaus plexippus*)

Brice X. Semmens[1], Darius J. Semmens[2], Wayne E. Thogmartin[3], Ruscena Wiederholt[4], Laura López-Hoffman[4], Jay E. Diffendorfer[2], John M. Pleasants[5], Karen S. Oberhauser[6], and Orley R. Taylor[7]

[1]Scripps Institution of Oceanography, University of California, San Diego, 9500 Gilman Drive, La Jolla CA 92093, USA, semmens@ucsd.edu

[2]United States Geological Survey, Geosciences and Environmental Change Science Center, Denver, CO 80225, USA, jediffendorfer@usgs.gov, dsemmens@usgs.gov

[3]United States Geological Survey, Upper Midwest Environmental Sciences Center, 2630 Fanta Reed Road, La Crosse, WI 54603, USA, wthogmartin@usgs.gov

[4]School of Natural Resources and the Environment and Udall Center for Studies in Public Policy, The University of Arizona, Tucson, AZ 85721, USA, rwiederholt@email.arizona.edu, lauralh@email.arizona.edu

[5]Department of Ecology, Evolution, and Organismal Biology, Iowa State University, Ames, IA 50011, USA, jpleasan@iastate.edu

[6]Department of Fisheries, Wildlife and Conservation Biology, University of Minnesota, St Paul, MN, USA

[7]Department of Ecology and Evolutionary Biology, University of Kansas, Lawrence, KS, USA

*Correspondence to: Brice X. Semmens, Scripps Institution of Oceanography, University of California, San Diego, 9500 Gilman Drive, La Jolla CA 92093, USA, semmens@ucsd.edu. Phone: 858-822-0518

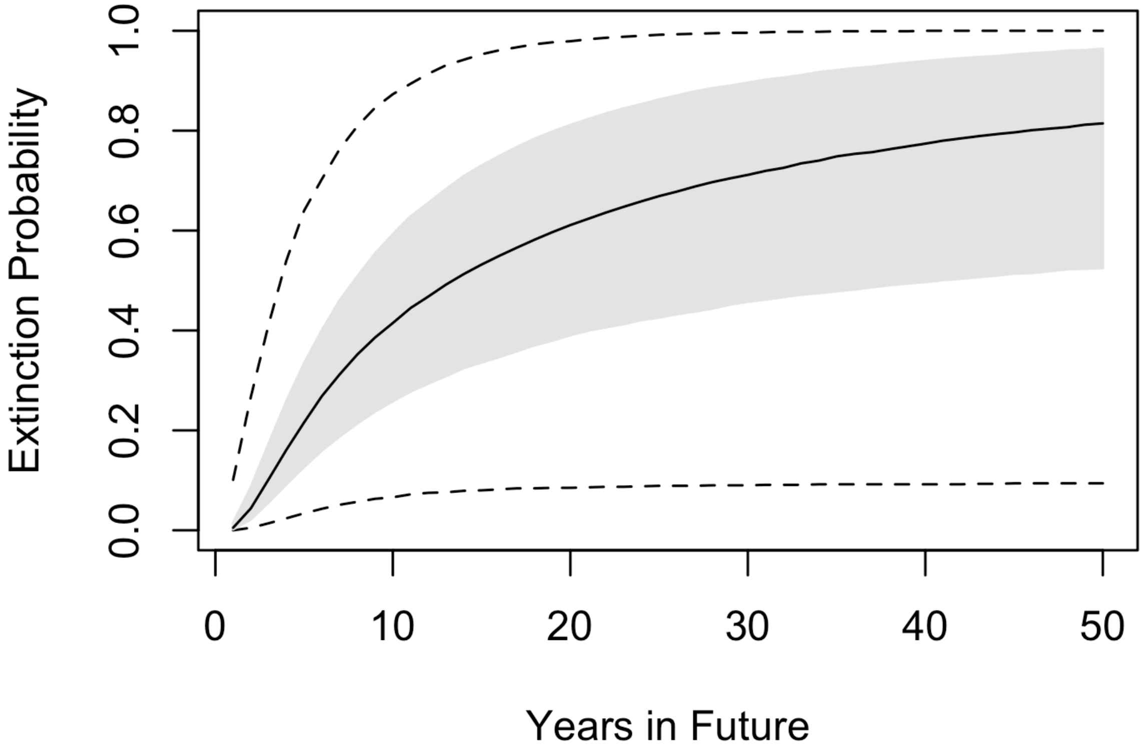

Extended Data Figure 1 | Extinction probability over 50 years for an extinction threshold of 0.25 ha. Figure depicts the median (solid line), 50% credible interval (shaded area), and 95% credible interval (dashed lines) monarch butterfly extinction risk estimates over the next 50 year based on a threshold of 0.25 ha. Estimates were calculated based on 1000 forward simulations of each set of MCMC posterior parameter draws resulting from our multivariate state-space monarch population model.

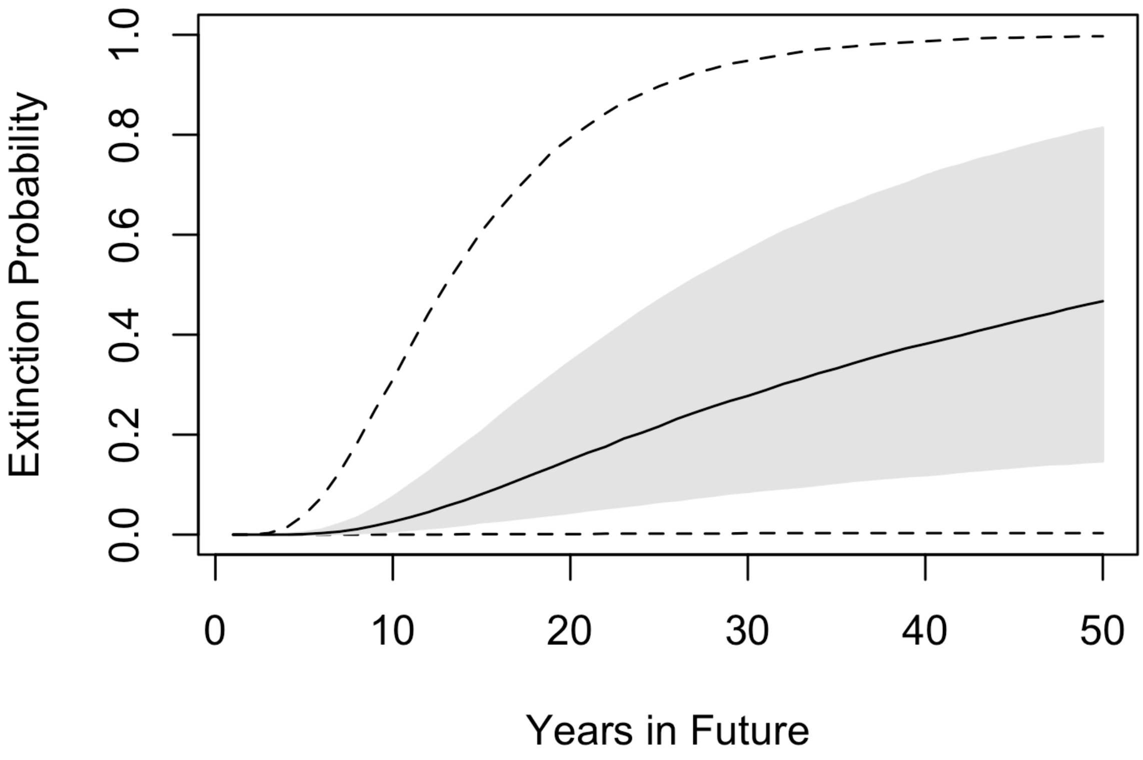

Extended Data Figure 2 | Extinction probability over 50 years for an extinction threshold of 0.01 ha. Figure depicts the median (solid line), 50% credible interval (shaded area), and 95% credible interval (dashed lines) monarch butterfly extinction risk estimates over the next 50 year based on a threshold of 0.01 ha. Estimates were calculated based on 1000 forward simulations of each set of Markov Chain Monte Carlo (MCMC) posterior parameter draws resulting from our state-space monarch population model.

Extended Data Table 1 | Time series data on the monarch population.

Year	Observed OW Area (ha)	Model Estimated OW Area (CI)	Production
**1993**	6.23	6.75 (3.32-14.60)	
**1994**	7.81	8.14 (4.28-16.07)	
**1995**	12.61	11.89 (5.98-22.16)	
**1996**	18.19	13.95 (7.09-27.12)	
**1997**	5.77	6.83 (3.66-13.90)	
**1998**	5.56	5.98 (3.19-11.62)	
**1999**	8.97	6.96 (4.73-10.41)	155,615,590
**2000**	2.83	5.16 (3.34-7.54)	94,534,475
**2001**	9.36	6.96 (4.82-10.56)	152,865,072
**2002**	7.54	5.04 (3.54-8.97)	58,626,900
**2003**	11.12	5.27 (3.55-10.93)	63,076,925
**2004**	2.19	2.94 (2.01-4.23)	23,815,429
**2005**	5.91	4.14 (2.88-6.68)	42,416,605
**2006**	6.87	4.58 (3.17-7.60)	52,417,747
**2007**	4.61	4.17 (2.82-6.26)	48,335,959
**2008**	5.06	3.35 (2.36-5.57)	24,404,080
**2009**	1.92	2.50 (1.68-3.69)	17,450,170
**2010**	4.02	3.72 (2.49-5.31)	42,737,180
**2011**	2.89	3.19 (2.07-4.54)	33,063,557
**2012**	1.19	2.08 (1.13-3.00)	14,449,400
**2013**	0.67	1.62 (0.68-2.43)	9,302,087
**2014**	1.13	2.22 (1.00-3.33)	19,902,386

Observed overwintering (OW) colony area, model estimated OW colony area (after accounting for observation error; median and 95% credible interval estimates) and Midwest larval production time series data used in the Bayesian state-space model.

Extended Data Table 2 | Parameter priors and estimates.

Parameter	Prior Distribution	Median and 95% CI
$\bar{u}$	~dunif(-10,10)	-0.061 (-0.378-0.253)
q	~dunif(0.001,10)	0.691 (0.529-0.899)
p	~dbeta(1,1)	0.933 (0.542-0.997)
$q * p$	--	0.600 (0.476-0.813)
a	~dunif(-10,10)	6.299 (6.039-6.545)
r	~dunif(0.001,10)	0.212 (0.192-0.053)
x_{1993}	~dunif(0,100)	6.750 (3.329-14.602)

Median and 95% credible interval estimates of parameters used in the multivariate auto-regressive state space model of monarch population dynamics. The terms *q*, *q*p*, and *r* are standard deviations. Note that the term q*p is a derived quantity from the two separately estimated parameters *p* and *q*. We duplicate x_{1993} from Extended Data Table 1 here because it is the only annual model estimated OW colony area that required the specification of a prior distribution.
